# Roles of caregiver-child interaction on the association of socioeconomic status with early childhood development: a population-based study in rural China

**DOI:** 10.1186/s12889-024-18803-4

**Published:** 2024-06-17

**Authors:** Rui Chang, Chunan Li, Mengna Wei, Yanfen Jiang, Jianduan Zhang

**Affiliations:** 1 Department of Maternal and Child Health, School of Public Health, Tongji Medical College, Huazhong University of Science and Technology, Wuhan, China; 2 Division of Child Healthcare, Department of Pediatrics, Tongji Hospital, Tongji Medical College, Huazhong University of Science and Technology, Wuhan, China

**Keywords:** Early childhood development, Neurodevelopment, Social-emotional development, Socioeconomic status, Caregiver-child interaction

## Abstract

**Objective:**

Socioeconomic status (SES) has been previously associated with children’s early development, health, and nutrition; however, evidence about the potential role of caregiver-child interaction in such associations was limited. This study aimed to explore the effect of caregiver-child interaction on the associations of SES with child developmental outcomes, including early neurodevelopment and social-emotional behavior.

**Methods:**

A cross-sectional survey was conducted among 2078 children aged 0–6 in a rural county that just lifted out of poverty in 2020 in Central China. The Ages & Stages Questionnaires-Chinese version (ASQ-C) and the Social-Emotional (ASQ: SE) questionnaire were used to assess children's early neurodevelopment and social-emotional behavior, respectively. Caregiver-child interaction was evaluated with the Brigance Parent–Child Interactions Scale. Regression-based statistical mediation and moderation effect were conducted with the PROCESS macro of SPSS.

**Results:**

Children with low SES had an increased risk of suspected neurodevelopmental delay [OR = 1.92, 95% *CI*: 1.50, 2.44] and social-emotional developmental delay [OR = 1.31, 95% *CI:* 1.04, 1.66]. The caregiver-child interaction partially mediated the associations of SES with child developmental outcomes; the proportion of the indirect effect was 14.9% for ASQ-C total score and 32.1% for ASQ: SE score. Moreover, the caregiver-child interaction had a significant moderation effect on the association of SES with ASQ-C total score (*P* < 0.05). A weaker association was observed in children with high-level caregiver-child interaction than in medium and low ones. Similar moderating effects were found among boys but not girls.

**Conclusion:**

Caregiver-child interaction plays a vital role in the relationship between SES and child development. Children with low SES households will benefit more in terms of their early development from intervention programs strengthening caregiver-child interaction.

**Supplementary Information:**

The online version contains supplementary material available at 10.1186/s12889-024-18803-4.

## Introduction

Early childhood is a sensitive period in life with rapid cognitive, physical, language, social and emotional development [1]. Early childhood development (ECD) lays the foundation for an individual’s health, well-being, and success throughout their life [2]. Adversity exposures in early childhood, from economic hardship to an unfavorable family environment, might interfere with the development processes in the early years [3]. Early childhood development has become a priority for the twenty-first century [4], and the World Health Organization also emphasizes that all children, especially those living in disadvantaged situations, require high-quality nurturing care to ensure they reach full potential and optimal development [5].

Including economic hardship and unfavorable family nurturing environment, those adversities have wide and long lasting effects on childhood and/or adult health and wellbeing [3]. Available evidence from low-income and middle-income countries suggests that children’s early exposure to poverty and other adversities is closely associated with deficits in their subsequent cognitive and social-emotional development, educational performance, adulthood income, and risk of chronic diseases [6, 7]. Neuroscience evidence indicates low socioeconomic status (SES) is associated with children’s smaller hippocampal grey matter volume [8] and this changed brain structure might mediate the relationships between poverty and children’s low cognitive, academic, and behavioral performance [9]. An estimation indicated that over 250 million children under five might never reach their full developmental potential due to low SES and poor nurturing care environment, and about 45 million of these children reside in China, ranked second globally [2, 10]. As one of the most important determinants of child development, SES has been documented to interpret inequalities, it has been shown that low family SES causes a higher incidence of developmental delay for children [11, 12]. Household SES is a measure of a family’s relative social position, which is best characterized by family income, parental education, and occupation as a whole rather than by any of them alone [13]. A population-based study assessed the association between maternal education and family income separately with the development of children [14]. However, previous studies often only use family income and few studies have examined the association between the SES index and early childhood development in China.

Moreover, nurturing care is also positively associated with children’s health, growth, and development. According to the WHO Nurturing Care Framework, the nurturing care is characterized by a home environment that is sensitive to children’s good health, adequate nutrition, responsive care-giving, opportunities for early learning, and security and safety [15]. Evidence was consistent in showing that inadequate learning resources and less interactive parenting activities are associated with children’s developmental delay in rural areas or households with deficient resources [16, 17]. A survey with a representative sample in Yunnan, one of the poorest provinces in China, showed that 72% of caregivers had not played with children and 47% had not read to them, which was more severe in left-behind children. On average, rural children play alone for about 2.5 h per day, implying the absence of caregiver-child interaction in the family [18]. Starting from early childhood, high-quality of caregiver-child interaction such as smiling, touching, talking, storytelling, listening to music, sharing and reading books, and engaging in play, builds neural connections that strengthen the child brain development [19]. However, the nurturing care in terms of responsive and emotional supportive, and developmentally stimulating and opportunities for play are often overlooked, especially in the least-developed rural areas. In China, more than a half of the caregivers in rural China had not interaction activities (e.g., playing or reading) with their children aged 0–35 months [18]. Inadequate stimulations or activities in early childhood affect brain development and increase the risk of development delay [20]. More importantly, neuroscientific evidence suggests that it is not poverty itself such as low SES that adversely affects child’s brain structure and development, but rather the effect poverty has on the parent/caregiver interaction relationship with the child [8, 21], suggesting an association of low SES with poor nurturing care style. In short, despite the relationships between household SES and child development, nurturing care style and child development, and household SES and nurturing care style have been independently examined in previous study, the mechanisms underlying these associations are hardly understood. Therefore, we hypothesize that caregiver-child interaction, as one of essential components of family nurturing care environment, play a mediation role in the association of household SES and early childhood neurodevelopment and social-emotional development.

Over the past 70 years, China has made remarkable achievements in terms of the Millennium Development Goals (MDGs), especially in the survival development goals of reducing child mortality [22]. Meanwhile, the Sustainable Development Goals (SDGs) have extended the focus from achieving child survival goals to thriving goals of high-quality care and early development. In China, although the government announced the eradication of extreme poverty in its last poor counties in 2020 [23], there still exist socioeconomic inequalities across regions and populations, and large numbers of disadvantaged populations and rural–urban disparities remain in the early development of children. For instance, data from several poor countries in Shannxi Province suggested that development delay among children increased from 13.4% when they were six months old to 50.4% when they were 30 months old [24], much higher than the prevalence reported in urban areas [25]. Although poverty alleviation has been achieved on a national scale, the socioeconomic inequality persists, it remains significant to investigate the child development status of post-poverty elevation areas, and the impact of households' SES on child development. If no interventions are conducted in an effective manner, a greater risk of inability to reach developmental potential among vulnerable individuals in resource-deficient households and a long-term compromise on the achievement of poverty eradication in China will inevitably arise.

In this study, we first proposed testing the associations between SES and ECD outcomes, including early neurodevelopment and social-emotional behavior among children aged 0–6 years in a county that alleviated absolute poverty in Central China. Furthermore, we assessed the mediating and moderating roles of caregiver-child interactions in those associations. Our objectives were to provide evidence that can be used to enhance policy formulation and program design to boost early childhood development in rural China and other regions outside of China with a similar situation.

## Methods

### Participants

This cross-sectional study was conducted in Xiaochang, one of the impoverished counties that just lifted out of poverty in 2020 in Hubei province in Central China. Using a stratified cluster sampling strategy, twelve towns in this county were divided into three layers according to the level of economic development. We randomly selected one town from each layer, and then several villages were randomly selected from each town. A total of 103 natural villages were included. The children aged 0 ~ 6 years who lived in these villages for more than one year and had no congenital anomaly were recruited. The information on household socioeconomic status and caregiver-child interaction were obtained through face-to-face interview questionnaire survey among the primary caregivers of children by professionally trained investigators, and the early childhood developmental outcomes, including neurodevelopment and social-emotional development, were assessed one-on-one among children by a professionally trained staff. Finally, 2078 children aged 0 ~ 6 years and their corresponding primary caregivers were included in the analysis, and the effective response rate of the questionnaire was more than 99.0%.

### Household socioeconomic status

Household SES is a measure of a family’s relative social position, which is best characterized by income, education, and occupation as a whole rather than by any of them alone. Therefore, in this study, the SES measure is based on five equally weighted, standardized components of family income, father’s education, mother’s education, father’s occupation, and mother’s occupation. Parental education was coded into three categories (≤ 9 years, 10–12 years, > 12 years); parental occupation was classified into three groups (group 1: farmers, laborers, and unemployed people; group 2: business and service workers, office clerks, and soldiers; group 3: professionals, technical personnel, and managers); family income was comprised of four categories (< 2000, 2000–4000, 4000–6000, ≥ 6000 RMB). The category score of parental education, occupation, and family income referenced the manual for scoring socioeconomic status by Lawrence W, and the SES score was measured based on the formula, SES score = 0.4*family income + (0.7*maternal education + 0.4*maternal occupation + 0.7*paternal education + 0.4*paternal occupation)/2 [26]. Finally, the SES was classified into three levels according to the SES score distribution in the study population. SES score ≤ 33rd percentile was defined as low SES, 33th-66th percentile was medium SES, and > 66th percentile was high SES.

### Caregiver-child interaction

Caregiver-child interaction was assessed by the Brigance Parent–Child Interactions Scale (BPCIS). The BPCIS included 18 statements on parent–child activities and parent perceptions of parenting with the Cronbach's α coefficient being 0.8, such as “I help my child learn by talking and showing him or her new things”, “I talk with my child when feeding or eating with him or her”, “I play with my child and show him or her things about toys” [27]. Parents are offered five response options, i.e., never, rarely, sometimes, often, or always, scoring 1, 2, 3, 4 and 5 respectively, with a higher total score represents higher-quality of parent–child interaction. In our study, the Cronbach's α coefficient of BPCIS was 0.83, indicating good internal consistency. Due to the number of migrant parents in rural areas, the parent–child interaction in this scale was extended to the primary caregiver-child interaction in current study.

### Early childhood development

In the current study, child developmental outcomes included children’s neurodevelopment and social-emotional behavior, evaluated using the Ages & Stages Questionnaires-Chinese version (ASQ-C) and Social-Emotional (ASQ: SE), respectively. The ASQ-C includes 21 questionnaires designed to screen and monitor the development of children aged 1 ~ 66 months, which has been documented a good reliability and validity in Chinese children, with the Cronbach's α coefficient of ASQ-C was 0.8, and the sensitivity and specificity for identifying developmental delay was 87.5% and 84.5%, respectively [28]. The ASQ-C contains five developmental domains such as communication (CM), gross motor (GM), fine motor (FM), problem-solving (CG), and personal-social (PS); each domain consists of six items. The answer is scored as 10, 5, and 0 points for each item. The score on each of the six items was summed to obtain a domain score, and each domain score was summed to obtain the ASQ-C total score, with lower scores representing poorer developmental outcomes. The ASQ-C domain scores below the cutoff point are considered suspected developmental delay (SDD) in that domain, and a suspected developmental delay in any of the above five domains was defined as the total suspected developmental delay [29]. The ASQ: SE is a brief parent/caregiver-reported instrument designed to screen the social-emotional developmental delay in children aged 3 ~ 66 months. The ASQ: SE comprised eight questionnaire forms for children of different ages. Each item had three response options (rarely or never, sometimes, most of the time) which were scored as 0, 5, 10, and also had a possible additional five scores if this specific behavior worries the parent/caregiver. The item scores add the additional worried scores were the total ASQ: SE scores. The total score beyond the cutoff score was identified as social-emotional developmental delay (SEDD). The validation of ASQ: SE indicated that it could serve as a good starting point for screening for social-emotional behavior problems among Chinese children, with the Cronbach's α coefficient being 0.7 [30].

### Covariates

Covariates included child gender (boy or girl), age (< 12, 12 ~ 36, or ≥ 36 months), birth weight, preterm (yes or no), delivery mode (vaginal delivery or cesarean section), only child (yes or no), feeding mode in the first six months (basic breastfeeding, mixed feeding or artificial feeding), left behind status (yes or not). Left-behind children were those whose fathers or mothers went out to work for more than six months.

### Statistical analyses

**Fig. 1:**
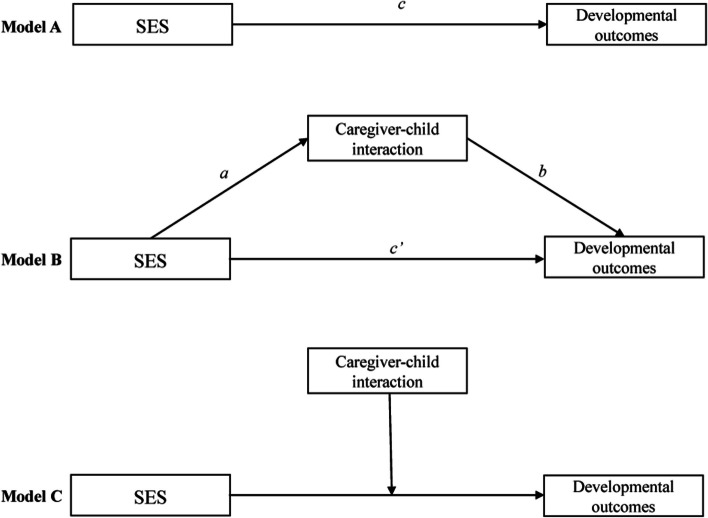
The hypothesis models in this study

Category variables were described in frequency and proportion [n (%)]. First, the univariate analysis of developmental delay was assessed using the chi-squared test. Second, a multinomial logistic regression model was performed to explore the association between SES and child developmental delay after controlling for the covariates (e.g., child age, sex, birth weight, gestational age, delivery mode, child number, feeding mode in the first six months, and left-behind status). The results were displayed with an odds ratio (OR) and a 95% confidence interval (95% *CI*). Third, multiple linear regression analyses were used to determine the associations between SES and developmental outcomes, and the *c* path coefficients represents the total effect (model A in Fig. 1). The SES was the independent variable and the developmental outcomes (e.g., total ASQ-C score, five domains score, or ASQ: SE score) were the dependent variable in the multiple linear regression analyses. Fourth, the mediation and moderation analyses were conducted in PROCESS using least squares regression. The simple mediation effect means that the effect of the independent variable (i.e. SES) on the dependent variable (i.e. developmental outcomes) acts through an intermediate variable (i.e. caregiver-child interaction), and all of these variables were conducted as continuous variables. In our study, caregiver-child interaction acts as the mediator (model B in Fig. 1) or moderator (model C in Fig. 1), the letters *a, b,* and *c’* represent path coefficients. The *a* path coefficient represents the effect of SES on caregiver-child interaction, and the *b* path coefficient represents the effect of caregiver-child interaction on the developmental outcomes; the *a and b* path coefficient constitute the indirect (mediating) effect. The *c’* path coefficient represents the effect of the SES on the developmental outcomes after controlling for the caregiver-child interaction, i.e. the direct effect. Thus, the total effect is equal to the direct effect plus the indirect effect (*c* = a*b + *c’*). Mediating effect analysis is to test whether the a*b effect exists and its proportion in the total effect, which indicating the degree of mediating effect. Finally, the bootstrap method was used to test the significance of mediating and moderating interaction effects. The 95% *CI* was estimated by the bias-corrected bootstrapping procedure, with the number of iterations set to 5,000. All data analyses were conducted using SPSS 26.0. The threshold of significance was defined as *P* < 0.05.

### Human ethics

Our study was approved by the Ethics Committees of the Tongji Medical College, Huazhong University of Science and Technology. All guardians of the children participating the current study signed informed consent.

## Results

### Summary of the descriptive information

Table 1 shows the basic characteristics of the participants. A total of 2078 participants (1,160 boys) were included in the current analysis. About 35% of children were younger than three years of age, more than half of children were cesarean Sect. (67.5%), not only child (64.0%), mixed feeding or artificial feeding (57.3%), and being left behind by one or both parents (70.4%). It is worth mentioning that 25.5% (529/2078) of children were taken care by their grandparents rather than one of parents. The percentage of parents with fewer than 12 years of education was nearly 90.0%, and the occupation with relatively low prestige (e.g., unemployed, farmer, laborer) was almost 70.0%. Most of the family’s monthly income was less than 628.6 USD (58.0%).

**Table 1:** Basic characteristics of the participants

Variable	Total (*N* = 2078)		Boy (*N* = 1160)		Girl (*N* = 918)	
	n	%	n	%	n	%
Child age (months)						
< 12	172	8.3	98	8.4	74	8.1
12 ~ 36	531	25.6	303	26.1	228	24.8
≥ 36	1364	65.6	752	64.8	612	66.7
Preterm						
Yes	167	8.0	98	8.4	69	7.5
No	1911	92.0	1062	91.6	849	92.5
Delivery mode						
Vaginal delivery	675	32.5	361	31.1	314	34.2
Caesarean section	1403	67.5	799	68.9	604	65.8
Only child						
Yes	749	36.0	418	36.0	331	36.1
No	1329	64.0	742	64.0	587	63.9
Left behind						
Yes	1463	70.4	794	68.4	669	72.9
No	615	29.6	366	31.6	249	27.1
Feeding mode in the first 6 months						
Basic breastfeeding	887	42.7	490	42.2	397	43.2
Mixed feeding	907	43.6	509	43.9	398	43.4
Artificial feeding	284	13.7	161	13.9	123	13.4
Maternal education (years)						
≤ 9	1521	73.2	860	74.1	653	71.1
10 ~ 12	339	16.3	190	16.4	157	17.1
> 12	218	10.5	110	9.5	108	11.8
Paternal education (years)						
≤ 9	1486	71.5	843	72.7	651	70.9
10 ~ 12	347	16.7	177	15.3	162	17.6
> 12	245	11.8	140	12.1	105	11.4
Maternal occupation						
Group 1	1424	68.5	785	67.7	639	69.6
Group 2	457	22.0	267	23.0	190	20.7
Group 3	197	9.5	108	9.3	89	9.7
Paternal occupation						
Group 1	1379	66.4	754	65.0	625	68.1
Group 2	467	22.5	273	23.5	194	21.1
Group 3	232	11.2	133	11.5	99	10.8
Household monthly income (USD)						
< 314.3	459	22.1	251	21.6	208	22.7
313.3 ~	746	35.9	427	36.8	319	34.7
628.6 ~	444	21.4	247	21.3	197	21.5
≥ 943.0	429	20.6	235	20.3	194	21.1

### Association of SES with child developmental outcomes

Table 2 shows the univariate analysis results, which revealed that children with low SES had the highest prevalence of SDD and SEDD (41.2% and 42.1%, respectively). Conversely, high SES children had the lowest prevalence of developmental delay (26.0% and 34.8%, respectively). Boys in low SES families had a higher prevalence of SDD than girls (45.5% vs. 35.6%, *P* < 0.05), while the prevalence of SEDD did not observe the significant difference.

**Table 2:** The prevalence of developmental delay at different SES levels (%)

Variable	SES	N	SDD	CM	GM	FM	CG	PS	SEDD
Overall	Low	713	41.2	14.6	11.8	16.5	15.8	22.3	42.1
	Medium	660	31.7	11.1	7.0	9.4	10.5	17.9	44.3
	High	689	26.0	7.4	5.8	8.9	6.8	15.4	34.8
	Total	2062	33.1	11.1	8.2	11.7	11.1	18.6	40.4
	χ^2^		37.70	18.38	18.62	25.05	29.33	11.39	13.76
	*P*		0.00	0.00	0.00	0.00	0.00	0.00	0.00
Boy	Low	404	45.5	17.1	11.4	18.8	18.8	26.0	42.8
	Medium	366	33.3	12.3	7.1	8.5	10.9	17.8	46.5
	High	381	27.8	7.1	5.2	8.7	6.3	16.3	34.0
	Total	1151	35.8	12.3	8.0	12.2	12.2	20.2	41.1
	χ^2^		28.21	18.21	10.61	25.76	29.5	13.42	12.71
	*P*		0.00	0.00	0.01	0.00	0.00	0.00	0.00
Girl	Low	309	35.6	11.3	12.3	13.6	12.0	17.5	41.2
	Medium	294	29.6	9.5	6.8	10.5	9.9	18.0	41.5
	High	308	23.7	7.8	6.5	9.1	7.5	14.3	35.9
	Total	911	29.6	9.5	8.6	11.1	9.8	16.6	39.5
	χ^2^		10.47	2.23	8.35	3.30	3.55	1.79	2.57
	*P*		0.01	0.32	0.01	0.19	0.16	0.40	0.27

*SES* Socioeconomic status, *SDD* suspected developmental delay, *CM* communication, *GM* gross motor, *FM* fine motor, *CG* problem-solving, *PS* personal-social, *SEDD* social-emotional developmental delay

Table 3 shows the association of SES with child developmental delay after adjusting for the confounders. As compared to the children with high SES, children with low SES had the highest risk of SDD [OR = 1.92, 95% *CI*: 1.50, 2.44] and highest risk of five domains delay, and had a higher risk of SEDD [OR = 1.31, 95% *CI:* 1.04, 1.66]; those results were also found among boys. Among girls, low SES was associated with the highest risk of SDD [OR = 1.70, 95% *CI*: 1.16, 2.50], but not related to the risk of any five domains delay or the SEDD risk compared with the high SES ones.

**Table 3:** The association between SES and child developmental delay

Variable	SES	SDD	CM	GM	FM	CG	PS	SEDD
Overall^a^	High	1	1	1	1	1	1	1
	Medium	1.33(1.04,1.71)*	1.52(1.03,2.24)*	1.31(0.83,2.06)	1.02(0.69,1.50)	1.63(1.10,2.44)*	1.19(0.89,1.61)	1.44(1.14,1.82)**
	Low	1.92(1.50,2.44)**	1.94(1.34,2.81)**	2.04(1.35,3.08)**	1.84(1.30,2.60)**	2.50(1.72,3.64)**	1.49(1.12,1.99)**	1.31(1.04,1.66)*
Boy	High	1	1	1				
	Medium	1.29(0.93,1.78)	1.75(1.04,2.93)*	1.52(0.81,2.84)	0.96(0.57,1.62)	1.96(1.13,3.38)*	1.06(0.71,1.57)	1.64(1.20,2.23)**
	Low	2.04(1.49,2.80)**	2.46(1.50,4.04)**	2.28(1.27,4.06)**	2.22(1.40,3.15)**	3.54(2.12,5.89)**	1.65(1.14,2.39)*	1.38(1.02,1.88)*
Girl	High	1	1	1	1	1	1	1
	Medium	1.40(0.95,2.06)	1.23(0.68,2.21)	1.10(0.57,2.13)	1.09(0.61,1.93)	1.30(0.72,2.36)	1.44(0.91,2.27)	1.23(0.86,1.74)
	Low	1.70(1.16,2.50)*	1.30(0.73,2.29)	1.79(0.98,3.25)	1.31(0.76,2.27)	1.52(0.85,2.69)	1.25(0.79,1.99)	1.24(0.87,1.76)

*SES* Socioeconomic status, *SDD* suspected developmental delay, *CM* communication, *GM* gross motor, *FM* fine motor, *CG* problem-solving, *PS* personal-social, *SEDD* social-emotional developmental delay

^*^represent *P* < 0.05, **represent *P* < 0.01

^a^Adjusted for child age, gender, birthweight, preterm, delivery mode, only child, feeding mode in the first 6 month, and left-behind status

### Mediation role of caregiver-child interactions

Table 4 shows the mediation effects of caregiver-child interaction on the association between SES and child developmental outcomes. After adjusting for confounders, the SES was positively associated with the ASQ-C total score and in all five domains scores but negatively associated with ASQ: SE score (*P* < 0.05). The mediation analysis found that SES positively associated with caregiver-child interaction (*P* < 0.05); caregiver-child interaction was positively associated with the ASQ-C total score and the scores in CM, FM, CG, and PS domains but was negatively associated with the ASQ: SE score (*P* < 0.05). The bootstrap test indicated significant mediating effects of caregiver-child interaction on the associations between SES and child developmental outcomes, except for the GM domain. Besides, the associations between SES and child developmental outcomes were still significant, which indicated the partially mediating effects of caregiver-child interactions in these associations (*P* < 0.05). The proportion of the mediating effect of caregiver-child interactions was 14.9% for ASQ-C total score [indirect effect (boot 95%*CI*): 0.106 (0.054, 0.165)], 12.7% for the CM score [indirect effect (boot 95%*CI*): 0.024 (0.010, 0.040)], 23.8% for the FM score [indirect effect (boot 95%*CI*): 0.025 (0.010, 0.042)], 16.7% for the CG score [indirect effect (boot 95%*CI*): 0.030 (0.016, 0.046)], 14.9% for the PS score [indirect effect (boot 95%*CI*): 0.020 (0.007, 0.035)], and 32.1% for ASQ: SE score [indirect effect (boot 95%*CI*): -0.153 (-0.206, -0.106)]. The mediating effects of caregiver-child interactions in the associations of SES with ASQ-C total score and ASQ: SE score were also found among boys and girls [indirect effect(boot 95%*CI*), ASQ-C total score among boy: 0.087 (0.029, 0.162), girl: 0.103 (0.022, 0.199); ASQ:SE score among boy: -0.129 (-0.201, -0.070), girl: -0.194 (-0.283, -0.120)].

**Table 4:** The mediating effect of caregiver-child interaction on the association between SES and child developmental outcomes

Outcomes		Path coefficients				Indirect effect (a × b)	Proportion [(a × b)/c × 100%]	Boot 95%CI
		c	a	b	c’			
Overall^a^	Total ASQ-C	0.713**	0.193**	0.551**	0.606**	0.106	14.9%	0.054, 0.165
	CM	0.191**	0.193**	0.126**	0.166**	0.024	12.7%	0.010, 0.040
	GM	0.102**	0.194**	0.033	0.096**	0.006	–-	-0.005, 0.019
	FM	0.105**	0.194**	0.129**	0.079*	0.025	23.8%	0.010, 0.042
	CG	0.182**	0.194**	0.157**	0.152**	0.030	16.7%	0.016, 0.046
	PS	0.139**	0.194**	0.107**	0.118**	0.020	14.9%	0.007, 0.035
	ASQ-SE	-0.475**	0.195**	-0.783**	-0.322**	-0.153	32.1%	-0.206, -0.106
Boy	Total ASQ-C	0.786**	0.169**	0.515**	0.698**	0.087	11.1%	0.029, 0.162
	CM	0.204**	0.169**	0.122*	0.183**	0.020	9.8%	0.003, 0.041
	GM	0.130**	0.172**	0.044	0.122**	0.007	–-	-0.005, 0.023
	FM	0.098*	0.172**	0.146**	0.073	0.025	25.5%	0.006, 0.047
	CG	0.213**	0.172**	0.133**	0.190**	0.023	10.8%	0.007, 0.041
	PS	0.156**	0.172**	0.072	0.143**	0.124	–-	-0.003, 0.030
	ASQ-SE	-0.581**	0.174**	-0.738**	-0.452**	-0.129	22.2%	-0.201, -0.070
Girl	Total ASQ-C	0.522**	0.201**	0.512*	0.418*	0.103	19.7%	0.022, 0.199
	CM	0.136**	0.201**	0.106	0.114*	0.021	–-	-0.001, 0.046
	GM	0.063	0.201**	0.007	0.061	0.001	–-	-0.019, 0.023
	FM	0.102	0.201**	0.091	0.084	0.018	–-	-0.003, 0.044
	CG	0.119*	0.201**	0.173**	0.084	0.034	28.6%	0.013, 0.059
	PS	0.101*	0.201**	0.133*	0.074	0.027	26.7%	0.007, 0.052
	ASQ-SE	-0.333**	0.201**	-0.967**	-0.138	-0.194	58.3%	-0.283, -0.120

*ASQ-C* the Ages & Stages Questionnaires-Chinese version, *ASQ-SE* Social-Emotional, *SES* Socioeconomic status, *CM* communication, *GM* gross motor, *FM* fine motor, *CG* problem-solving, *PS* personal-social

^*^P < 0.05, ** *P* < 0.01

^a^Adjusted for child age, gender, birthweight, preterm, delivery mode, only child, feeding mode in the first 6 month, and left-behind status

### Moderation role of caregiver-child interaction

**Fig. 2:**
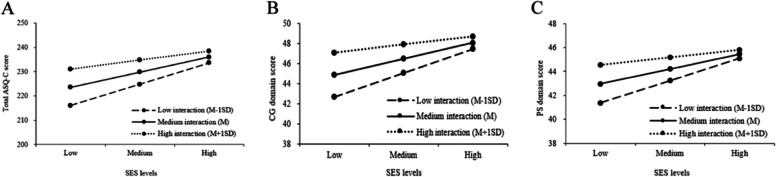
The simple regression lines of SES on child total ASQ-C score, CG, and PS domain score under different levels of caregiver-child interaction

Table 5 shows the moderation effect of caregiver-child interaction on the associations between SES and child developmental outcomes. After adjusting for confounders, the interaction effects of SES and caregiver-child interaction on the ASQ-C total score, CG, and PS score were significant (*P* < 0.05). As visualized in Fig. 2, the associations of SES with ASQ-C total score, CG, and PS score were weaker at a high level of caregiver-child interaction than at medium and low interaction. A similar moderation effect was observed in boys (Appendix).

**Table 5:** The moderating effect of caregiver-child interaction on the association between SES and child development outcomes

Outcomes	*β*	SE	*P*	Boot 95%CI
Total ASQ-C	-0.031	0.012	**0.013**	-0.056, -0.006
CM	-0.004	0.003	0.170	-0.011, 0.002
GM	-0.004	0.003	0.136	-0.010, 0.001
FM	-0.004	0.003	0.240	-0.011, 0.002
CG	-0.009	0.003	**0.003**	-0.016, -0.003
PS	-0.007	0.003	**0.028**	-0.014, -0.001
ASQ-SE	-0.003	0.008	0.640	-0.019, 0.012

Adjusted for child age, gender, birthweight, preterm, delivery mode, only child, feeding mode in the first 6 month, and left-behind status

*ASQ-C* the Ages & Stages Questionnaires-Chinese version, *ASQ-SE* Social-Emotional, *SES* Socioeconomic status, *CM* communication, *GM* gross motor, *FM* fine motor, *CG* problem-solving, *PS* personal-social

## Discussion

Our findings revealed that children in low SES households had a higher risk of early developmental delay; the prevalence of SDD and SEDD were 41.2% and 42.1% among children with low SES, respectively. Compared to the children in high SES households, the children with low SES families had the highest risk of SDD (*OR* = 1.92) and higher risk of SEDD (*OR* = 1.31). The positive association between SES and caregiver-child interaction was also significant (*P* < 0.05). Furthermore, high-quality caregiver-child interaction attenuates the adverse effects of low SES on child developmental delay, and the caregiver-child interaction partially mediated the association of SES with the child's early neurodevelopment and social-emotional development. We also found that caregiver-child interaction moderated the associations of SES with children’s neurodevelopment, especially in boys. Therefore, intervention programs strengthening caregiver-child interaction was urgently needed in rural China.

### Children in low SES households had a higher risk of early developmental delay

Although fewer than 6 million under-5 child deaths occur each year, about 43% of children in low- and middle-income countries (LMICs) are at risk of not reaching their developmental potential due to stunting and poverty alone [31]. A poor start in life limits children’s abilities to benefit from education, which may lead to long-term adverse effects such as lower productivity and social tensions. Consequences affect not only present but also future generations [5]. As a predictor of poverty, SES was associated with a wide range of health, cognitive, and socioemotional outcomes in children, with lasting effects from birth to adulthood [11]. In our study, we found that children in low SES households in rural China were more susceptible to early neurodevelopmental delay and social-emotional behavior problems, and the prevalence of SDD and SEDD reached 41.2% and 42.1%, respectively. Our findings were consistent with a cross-sectional survey of children aged 36–60 months in Iran that revealed the SES index could be used to interpret the inequalities of child development [11]. A prospective cohort study documented that low SES (e.g., maternal lower levels of education, low income, and poor housing conditions) is significantly correlated with child developmental delay [32]. The mechanisms linking SES to child development involve the differences in access to material and social resources or reactions to stress-inducing conditions by the children and their parents [11]. Low SES would increase the risk of unhealthy behaviors, inadequate nutrition, failure to access health care, maternal diseases, and drug abuse, consequently increasing child developmental delay [33]. Furthermore, children with low-income families are more exposed to family conflict, violence, separation, and instability, posing adverse effects on their development [34]. Although China has scored a complete victory in eradicating absolute poverty in 2020, there are still a large number of vulnerable individuals living with disadvantages and suffering from developmental delay risk. In the phase of rural revitalization in China, more emphasis should be attached to the development situation of children living in low SES families to consolidate the achievements of poverty alleviation. Although several ECD programmes, such as home visiting services and ECD centers establishment, have been launched by government departments or some other organizations, the coverage is minimum and the effectiveness remains unknown. Cost-effective ECD interventions should be further explored to narrow the children’s development inequality and inadequacy in rural–urban.

### High-quality caregiver-child interaction attenuates the adverse effects of low SES on child developmental delay

Determinants for development in early life can be found among biological and socioeconomic factors, and in stimulation and learning opportunities [35]. Evidence documented that stimulating caregiver-child interactions, including smiling, touching, talking, storytelling, listening to music, sharing and reading books, and engaging in play, are highly beneficial for early childhood development and have long-lasting positive effects [19]. Studies have emphasized a relationship between SES and children’s cognitive and linguistic learning through family simulations, the number of siblings, and the number of family members who live together [36]. Although the neuroscientific evidence suggests that poor household SES associated with smaller white and cortical gray matter and hippocampus and amygdala volumes in school aged children, it is not poverty itself that adversely effects brain development, but rather the effect poverty has on the parent/caregiver relationship with the child. Our study revealed that the caregiver-child interaction partially mediated the association of SES with the child's early neurodevelopment and social-emotional development. In other words, the adverse effects of low SES on child developmental outcomes could be attenuated by increasing caregiver-child interaction. Evidence showed that nutrition, home environment, child-parent interaction, and facilitating and stimulating learning experiences affect child development, and higher SES would lead to a better learning environment, while lower SES is a barrier to learning and accessing cognitive stimulation such as accessing newspapers, books, and toys [11]. Therefore, receiving stimulation and interaction during infancy and early childhood is vital to successful development [37]. Our study reinforced the importance of the quality of caregiver-child interaction on children’s early development in low SES families in rural China, providing ECD intervention tailored to our country.

### Intervention programs strengthening caregiver-child interaction was urgently needed in rural China

ECD intervention programmes have been launched worldwide as a vital effort to achieve the UN Sustainable Development Goals. Evidence revealed that investment in ECD benefits longer-term health, learning, and behavior [31]. In China, to ensure high-quality nurturing care in rural families and narrow the early development gap between urban and rural children, the government has developed a range of Early Child Development programmes since 2009. Children's nutrition status (e.g., stunting, underweight, and wasting) in poor rural China has been greatly improved after the nutrition supplement program called Ying Yang Bao across the country [38]. However, a shortage of early stimulation is a severe issue, especially in less-developed rural areas. A survey conducted in the least developed province of China indicated that more than two-thirds of caregivers have no interaction activities (e.g., play or reading) with their child [24]. Our study expanded the evidence that higher risk of developmental delay and poor nurturing environment among children aged 0–6 were still challenging in rural China even after eradicating absolute poverty across China. Moreover, our study suggested that opportunities for early learning, including adequate caregiver-child interaction, could attenuate the detrimental effects of low SES on child development. Low-cost activities, such as storytelling, singing, and playing with household objects, expose young children to experiences that promote early development [39]. Therefore, actions should be taken to improve caregivers’ awareness of the importance of daily interaction with their children, especially those caregivers with low SES.

Our study had several strengths and limitations. To our knowledge, this is the first study conducted in the context of poverty alleviation across China to evaluate the effect of caregiver-child interaction on the association of SES with child development, which provides solid evidence for future intervention research. Besides, the study population in our research was representative and covered from two months to six years old and included 103 villages. Moreover, our study simultaneously evaluated the child's early neurodevelopment and social-emotional development, which could comprehensively understand the development of rural children. However, certain limitations of this study should be recognized. First, because of the cross-sectional design in this study, we were not able to assess the causal associations of the nurturing environment (e.g., SES and caregiver-child interaction) with early childhood development, and a further cohort study is needed to demonstrate those relationships. However, our large random sample size and precise results provided several answers that can move our understanding of associations among the three variables. Second, as the self-reported data by the caregiver may lead to under-reporting, it’s inevitable for information bias to occur. Third, we do not assess the roles of caregiver-child interaction between parent migration and non-migration. Future research could consider this variable to better uncover the development situations of vulnerable children with migration parents.

## Conclusions

Our study found that SES was positively associated with early childhood neurodevelopment and social-emotional development in a Chinese county lifted out of poverty. The caregiver-child interaction plays an essential role in such associations. Intervention programs aiming to improve the early development of children in low SES families should strengthen the caregiver-child interaction.

### Supplementary Information

Supplementary Material 1.

## Data Availability

The datasets used and/or analysed during the current study are available from the corresponding author on reasonable request.
