# Supplementary material for: Roles of caregiver-child interaction on the association of socioeconomic status with early childhood development: a population-based study in rural China
Source: BMC Public Health. 2024 Jun 17;24:1604. doi: 10.1186/s12889-024-18803-4 (PMC11181537; doi:10.1186/s12889-024-18803-4)
Supplement: Supplementary file 1 — Supplementary Material 1. [file 12889_2024_18803_MOESM1_ESM.docx]

| **Supplement table 1 The moderating effect of caregiver-child interaction on the association between SES and child development outcomes among boys** | | | | | |
| --- | --- | --- | --- | --- | --- |
| Independent variables | Outcomes | *β* | SE | *P* | Boot 95%CI |
| SES * caregiver-child interaction | Total ASQ-C | -0.034 | 0.016 | **0.031** | -0.065, -0.003 |
|  | CM | -0.005 | 0.004 | 0.275 | -0.013, 0.004 |
|  | GM | -0.004 | 0.003 | 0.280 | -0.012, 0.003 |
|  | FM | -0.003 | 0.004 | 0.472 | -0.013, 0.006 |
|  | CG | -0.011 | 0.004 | **0.005** | -0.020, -0.003 |
|  | PS | -0.009 | 0.004 | **0.036** | -0.017, -0.001 |
|  | ASQ-SE | 0.001 | 0.010 | 0.904 | -0.019, 0.022 |


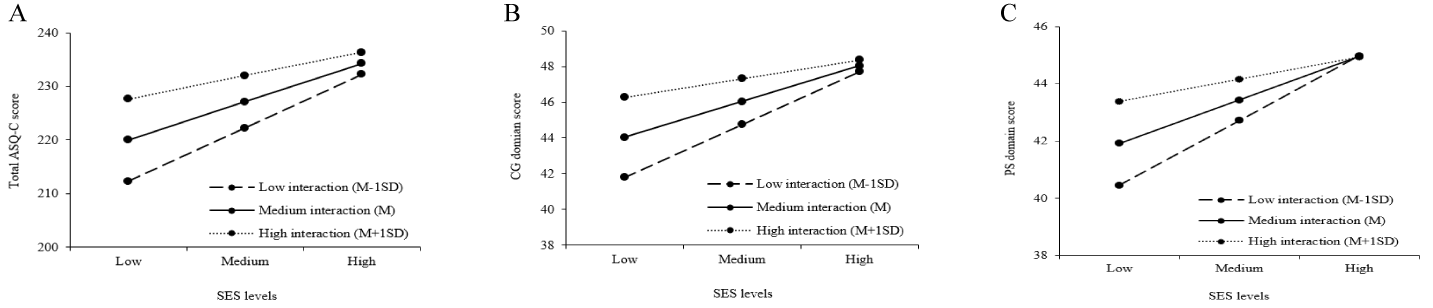


**Supplement figure 1. The simple regression lines of SES on child total ASQ-C score, CG and PS domain score under different levels of caregiver-child interaction among boy**

| **Supplement table 2 The moderating effect of caregiver-child interaction on the association between SES and child development outcomes among girls** | | | | | |
| --- | --- | --- | --- | --- | --- |
| Independent variables | Outcomes | *β* | SE | *P* | Boot 95%CI |
| SES * caregiver-child interaction | Total ASQ-C | -0.011 | 0.021 | 0.584 | -0.054, 0.030 |
|  | CM | -0.002 | 0.005 | 0.701 | -0.013, 0.008 |
|  | GM | -0.002 | 0.005 | 0.691 | -0.012, 0.008 |
|  | FM | -0.003 | 0.005 | 0.600 | -0.014, 0.008 |
|  | CG | -0.002 | 0.005 | 0.680 | -0.012, 0.008 |
|  | PS | -0.002 | 0.005 | 0.687 | -0.013, 0.008 |
|  | ASQ-SE | 0.001 | 0.013 | 0.954 | -0.026, 0.024 |
